# Factors associated with fasting time in pediatric patients hospitalized for surgery

**DOI:** 10.1590/0100-6991e-20253889-en

**Published:** 2025-11-24

**Authors:** LAURA NASPITZ, FERNANDA LUÍSA CERAGIOLI OLIVEIRA, RENATO LOPES DE SOUZA, TULIO KONSTANTYNER

**Affiliations:** 1 - Universidade Federal de São Paulo, Pediatria - São Paulo - SP - Brasil; 2 - Universidade Federal de São Paulo, Pós graduação em Nutrição - São Paulo - SP - Brasil

**Keywords:** Fasting, Preoperative Period, Postoperative Care, Surgical Procedures, Operative, Pediatrics., Jejum, Período Pré-Operatório, Cuidados Pós-Operatórios, Procedimentos Cirúrgicos Operatórios, Pediatria

## Abstract

**Introduction:**

Shorter fasting periods before and after surgery have been associated with better postoperative recovery and lower morbidity and mortality. However, it is not always possible to achieve current recommendations in pediatric practice. Therefore, it is essential to study fasting time and its associated factors to implement better care strategies.

**Methods:**

Cohort of 284 pediatric patients admitted for surgery between 2020-2021, Hospital São Paulo, Brazil. Data was collected through interviews and medical records. Simple and multiple linear models and logistic regression models were adjusted to study the associations.

**Results:**

All preoperative patients fasted for a prolonged period and most resumed feeding 6 hours after the end of anesthesia. Preoperative fasting time was shorter for elective surgery than for urgent surgery (p=0.025). Factors associated with a longer preoperative fasting time (minutes) were: older age in years (ß=10; 95% CI=5.2-14.8) and history of previous surgery (ß=76.6; 95% CI=28.0-125.1). Factors associated with postoperative fasting time longer than 6 hours were: no immediate postoperative care in the surgical ward (OR=6.05; 95%CI=2.25-16.22), presence of complications during surgery (OR=3. 53; 95%CI=1.19-10.47), major operation size (OR=3.85; 95%CI=1.49-9.93), abdominal surgery (OR=36.52; 95%CI=13.48-98.91) and vomiting in the first 24 hours (OR=3.44; 95%CI=1.54-7.69).

**Conclusion:**

There are potentially modifiable factors associated with longer fasting times. Education and organization of the healthcare team regarding patient characteristics, care dynamics, and clinical complications may contribute to greater optimization of fasting times in pediatric surgical patients.

## RESUMO

**Objetivo:**

O menor tempo de jejum para cirurgias tem sido associado a melhor recuperação pós-operatória e menor morbimortalidade. No entanto, nem sempre é possível alcançar as recomendações atuais na prática pediátrica. Sendo assim, é essencial conhecer o tempo de jejum e seus fatores associados para elaboração de estratégias assistenciais.

**Métodos:**

Coorte com 284 pacientes pediátricos admitidos para cirurgia entre 2020-2021, no Hospital São Paulo, Brasil. Dados foram obtidos por meio de entrevista e do prontuário médico. Para o estudo das associações foram ajustados modelos de regressão linear e logística simples e múltiplos.

**Resultados:**

Todos pacientes apresentaram jejum pré-operatório prolongado e a maioria reiniciou a dieta após 6 horas do término anestésico. O tempo de jejum pré-operatório para cirurgias eletivas foi menor do que para as de urgências (p=0,025). Os fatores associados ao maior tempo de jejum pré-operatório (minutos) foram: maior idade em anos (β=10; IC95%=5,2-14,8) e ocorrência de cirurgia anterior (β=76,6; IC95%=28,0-125,1). Os fatores associados ao tempo de jejum pós-operatório superior a 6h foram: não realização do pós-operatório imediato na enfermaria cirúrgica (OR=6,05; IC95%=2,25-16,22), presença de complicações durante a cirurgia (OR=3,53; IC95%=1,19-10,47), porte cirúrgico maior (OR=3,85; IC95%=1,49-9,93), cirurgia do tipo abdominal (OR=36,52; IC95%=13,48-98,91) e presença de vômitos nas primeiras 24 horas de pós-operatório (OR=3,44; IC95%=1,54-7,69).

**Conclusão:**

Há fatores potencialmente modificáveis que foram associados ao maior tempo de jejum. Treinamento e organização da equipe assistencial quanto às características dos pacientes, dinâmica do atendimento e intercorrências clínicas podem contribuir com maior adequação do tempo de jejum de pacientes pediátricos internados para procedimentos cirúrgico.

## INTRODUCTION

The first reports of the use of anesthetics for surgical procedures date back to the mid-nineteenth century. And since then, preoperative fasting has begun to be a concern^1^ ^,^ ^2^. Initially, the guidelines were less restrictive. However, after Mendelson’s work in 1946, reporting cases of pulmonary aspiration in obstetric patients, night fasting was instituted^3^. With the evolution of medicine, pulmonary aspiration during induction or during anesthesia has become rare in any age group, with an incidence of between 0.02% and 0.1% and morbidity and mortality close to zero^4^ ^,^ ^5^.

Surgery generates metabolic stress, with an increase in insulin resistance and the body’s inflammatory response, which are exacerbated by prolonged fasting, both before surgery and in the postoperative period. Longer fasting time is associated with a higher risk of unfavorable outcomes, such as a higher infection rate, suture dehiscence, metabolic ileus, greater need for fluid use, and length of hospital stay. Thus, reducing fasting time has gained importance for the success of surgical treatment^6^ ^,^ ^7^.

In this sense, national and international groups recommend preoperative fasting of two hours for clear liquids without residues, four hours for breast milk, six hours for milk formula, cow’s milk and light meals, and eight hours for fatty meals^8^ ^-^ ^11^. However, studies have shown that children can remain 16 to 18 hours without receiving any type of diet in the preoperative period^12^ ^,^ ^13^. 

Initiation of postoperative dietary intake is frequently delayed until resolution of adynamic ileus, despite evidence indicating that the early administration of small amounts of oral fluids can stimulate gastrointestinal function and expedite the attainment of caloric and protein requirements^6^ ^,^ ^13^ ^-^ ^16^.

There is growing interest in reducing length of hospital stay and postoperative complications in children and adolescents. Despite current recommendations for abbreviating perioperative fasting, it is not always possible to implement this strategy due to the operational difficulty associated with the dynamics and functioning of hospital care. In this sense, the knowledge about pre- and postoperative fasting procedure enables the elaboration of institutional protocols to adapt the medical prescription and, consequently, reduce complications and improve the quality of the service provided.

Thus, the objectives of the present study were to identify fasting time and its associated factors in pediatric patients hospitalized for surgical procedures.

## METHODS

### Design, population, and sample

The present study is an observational, contemporary, cohort study conducted in the pediatric surgery ward of Hospital São Paulo (São Paulo, São Paulo, Brazil), which is a university and public service. The study follow-up period was from the moment of patients’ admission to the ward until hospital discharge or up to 30 days of hospitalization.

The recruitment period was 14 months (01/08/2020 to 03/10/2021). The inclusion criterion was age between 28 days of life and 18 incomplete years, and the exclusion criteria were emergency surgeries, hospitalization time of less than 24 hours, preoperative or postoperative period occurring in another hospital service, patients who did not undergo surgical procedure, and readmission during the recruitment period.

The sample size was estimated at 300 patients, so that the 1:1 exposed and unexposed sample was sufficient to identify odds ratios of 1.97 and risk ratios of 1.42 (alpha = 0.05, beta = 0.20), with an estimated prevalence of the categorical endpoint studied (longer fasting time) in the unexposed group of 40%. For continuous evaluations, this sample calculation of 300 individuals was sufficient to estimate a correlation coefficient greater than or equal to 0.15, with a unilateral alpha of 0.05 and beta of 0.20.

### Data collection

Researchers who had received training in the measurement instruments and approach methods collected the information. An operational manual was developed and implemented to ensure consistency in fieldwork procedures. Epidemiological, clinical, ward, and operating room characteristics were documented at admission and during hospitalization. Data were obtained from electronic medical records, the surgical map, and interviews with parents or guardians. 

Interviews with parents or guardians were conducted within the first 24 hours following the children’s surgical procedures, thereby enhancing data quality and minimizing recall bias. To further reduce the likelihood of response bias, patients and their families were not informed of the primary objectives of this study

### Variable definitions

The anesthetic size was defined according to the guidelines published by the AMB (Brazilian Medical Association), the CFM (Federal Council of Medicine) and FENAM (National Federation of Doctors)17. Surgical size was classified by surgery duration as I (1 to 2 hours), II (2 to 4 hours), III (4 to 6 hours), and IV (over 6 hours).

Anesthetic risk was assessed using the American Society of Anesthesiology (ASA) classification^18^, while family socioeconomic status was determined according to the Brazilian Economic Classification by the Brazilian Association of Research Companies (ABEP)^19^.

Prolonged preoperative fasting was defined according to the type of last food ingested: more than two hours for clear liquids without residues, four hours for breast milk, six hours for milk formula, cow’s milk, and light meals, and eight hours for fatty meals.

Postoperative fasting longer than six hours was considered prolonged and the intraoperative complications evaluated were shock, arrhythmia, cardiorespiratory arrest, severe bleeding, hypoglycemia, hyperthermia, viscera injury, airway obstruction, and severe electrolyte disturbances. 

### Outcomes studied

- Preoperative fasting duration: The interval from the last meal, as recorded by guardians, to the initiation of anesthesia.
- Postoperative fasting time: The interval from anesthesia end to diet initiation (liquid, milk, or solid), as reported by guardians.
- Total fasting time: Sum of preoperative time, postoperative time, and total anesthetic time.

### Data analysis

The researchers checked the quantitative data for internal consistency before processing and analysis. One researcher entered the data into Excel, then verified and validated the content to correct errors.

Univariate and bivariate descriptive statistics were used to study associations. The data were presented as means with standard deviation, median with interquartile range, and prevalence with confidence interval.

The Mann-Whitney test compared medians, while simple linear regression assessed associations between continuous variables. Sample distribution was determined using Kolmogorov-Smirnov and Shapiro Wilk tests20. Associations with categorical variables were analyzed via simple logistic regression.

Preoperative fasting was studied continuously in minutes. To normalize the sample, we excluded eleven outliers (preoperative fasting of more than 1,440 minutes)^21^. Postoperative fasting was analyzed as either less than or greater than six hours. To control confounding variables on the effect of independent variables on the preoperative fasting time, we performed multiple linear regression with estimates of beta coefficients. In the same sense, for the postoperative fasting time, we applied multiple logistic regression with odds ratio (OR) estimates^22^.

In both multivariate analyses, variables with a p-value below 0.20 in the univariate analysis were selected for inclusion. The stepwise backward variable selection method was used, and variables were removed in order of the greatest reduction in statistical significance of the associations (p>0.05)^22^. The selection of explanatory and control variables for permanence in the final multiple models was based on their statistical association with the outcomes (p<0.05). In addition, control variables were included for estimates of independent effects. The statistical package used was STATA 14. The maximum level of 0.05 was chosen (error α maximum of 5%) to indicate a statistically significant association. 

### Ethical Aspects

In compliance with the determinations of Resolution 196/96 of the National Health Council and Resolution 466/12 of the Brazilian Ministry of Health, which determine the guidelines and regulatory standards for research involving human beings, this project was submitted to the analysis of the Ethics in Research Committee of the Federal University of São Paulo (CAAE: 30630920.8.0000.5505; Opinion No. 4,161,822). All data collection procedures occurred after the patients’ parents or guardians signed the Informed Consent Form. 

## RESULTS

The present study recruited and interviewed 305 individuals. Of these, 11 had some of the exclusion criteria. Of the remaining 294, ten (3.4%) were lost due to incomplete information on the outcomes studied. Thus, 284 made up the final sample.

Table 1 shows the sociodemographic characteristics and personal history of the patients studied. The sample consisted of 60.2% male patients, and 51.8% were aged 5 years or older.

**Table 1:** Sociodemographic characteristics and pathological and neonatal history of pediatric patients hospitalized and undergoing surgical procedures at Hospital São Paulo (August/2020 to October/2021).

Variable		N	% (Confidence Interval)
Sex	Female	113	39.8 (34.2-45.6)
	Male	171	60.2 (54.4-65.8)
Age	< 60 months	137	48.2 (42.4-54.1)
	³ 60 months	147	51.8 (45.9-57.6)
ABEP - Economy Class	A, B1 or B2	82	29.1 (24.0-34.7)
	C1, C2 or D-E	200	70.9 (65.3-76.0)
Maternal Age in years	Less than or equal to 19	7	2.5 (1.2-5.2)
	Greater than 19	272	97.5 (94.8-98.8)
Maternal schooling (years of study)	10 or less	93	33.8 (28.4-39.6)
	11 or more	182	66.2 (60.3-71.6)
Paternal Age in years	Less than or equal to 19	4	1.5 (0.6-3.9)
	Greater than 19	263	98.5 (96.1-99.4)
Paternal Education (years of study)	10 or less	97	40.8 (34.6-47.2)
	11 or more	141	59.2 (52.8-65.4)
Congenital pathological history	Yes	161	56.7 (50.8-62.4)
	No	123	43.3 (37.6-49.2)
Acquired pathological history	Yes	144	50.7 (44.9-56.5)
	No	140	49.3 (43.2-55.1)
Previous surgery	Yes	133	46.8 (41.1-52.7)
	No	151	53.2 (47.3-58.9)
Prematurity	Yes	58	21 (16.6-26.3)
	No	218	79 (73.7-83.4)
Birth weight less than 2500g	Yes	53	21.5 (16.7-27.1)
	No	194	78.5 (72.9-83.3)

. Table 2 presents information related to hospitalization. Elective surgeries corresponded to 68.7% of the sample, and of these, two thirds had a scheduled time on the surgical map. Most medical prescriptions for preoperative fasting, 59.5%, did not follow the recommendations of the pre-anesthetic evaluation form (APA). In addition, 77.9% of prescriptions of individuals hospitalized for elective surgery contained the guidance “night fasting from 11 pm” or “night fasting from midnight”, regardless of the scheduled start time for surgery and the type of last prescribed diet. Of the 195 preoperative prescriptions for elective surgeries, only eight (4.1%) presented any distinction in the recommended time between solid meal, clear liquid, or breast milk. 

**Table 2:** Clinical and care characteristics related to the pre-, intra-, and postoperative period of pediatric patients hospitalized and undergoing surgical procedures at Hospital São Paulo (August/2020 to October/2021).

Variable		N	% (Confidence Interval)
Preoperative hospital stay	In the surgical ward	227	79.9 (74.8-84.2)
	Other location	57	20.1 (15.8-25.2)
Elective surgery	Yes	195	68.7 (63-73.8)
	No	89	31.3 (26.2-37)
Scheduled time on the surgical map - electives only	Yes	134	68.7 (61.8-74.9)
	No	61	31.3 (25.1-38.2)
Time of fasting guidelines conveyance	Day before surgery	273	96.5 (93.5-98.1)
	Another moment	10	3.5 (1.9-6.5)
Manner of fasting guidelines conveyance	Personally	256	90.5 (86.4-93.4)
Variable		N	% (Confidence Interval)
	Other way	27	9.5 (6.6-13.6)
Fasting guidelines conveyor	Surgeon	144	50.9 (45-56.7)
	Other professional	139	49.1 (43.3-55)
Prescription of elective surgery fasting follows APA guidance	Yes	51	40.5 (32.2-49.4)
	No	75	59.5 (50.6-67.8)
Breakfast allowed for elective surgery in the afternoon	Yes	6	14 (6.2-28.5)
	No	37	86 (71.5-93.8)
Afternoon elective surgery with early start	Yes	16	37.2 (23.7-53)
	No	27	62.8 (47-76.3)
ASA Rating	I or II	223	90.3 (85.9-93.4)
	III, IV, V or VI	24	9.7 (6.6-14.1)
Anesthetic Size	0-4	155	54.6 (48.7-60.3)
	5-7	129	45.4 (39.7-51.3)
Surgical Size	1-2	234	82.7 (77.8-86.7)
	3-4	49	17.3 (13.3-22.2)
Intraoperative complications	Yes	30	10.6 (7.5-14.7)
	No	254	89.4 (85.3-92.5)
Immediate postoperative hospitalization site	In the surgical ward	249	87.7 (83.3-91)
	Other location	35	12.3 (9-16.7)
Vomiting in the first 24 hours postoperatively	Yes	75	26.4 (21.6-31.9)
	No	209	73.6 (68.1-78.4)

For elective surgeries with a scheduled time on the daily surgical map, delays in start of anesthesia of 60 minutes or more occurred in 96 cases (71.6%).

For surgeries scheduled in the afternoon, only 14% of patients were allowed to consume breakfast and in 37.2% of them the procedure start was earlier than scheduled on the surgical map. 

According to the established criteria, prolonged fasting occurred both in the preoperative and postoperative periods (Table 3). For the preoperative period, all patients remained fasting for longer than the current recommendations.

**Table 3:** Fasting time of patients admitted to the pediatric surgery ward of Hospital São Paulo (August/2020 to October/2021), according to the type of surgery.

	Median (IQR) minutes	Range minutes	Median (IQR) Hours: minutes	Range Hours: minutes
Fasting time - all patients				
Preoperative (n=283)	700 (590-920)	200-2,905	11:40 (9:50-15:20)	3:20-48:25
Postoperative (n=284)	220 (150-372)	5-5,110	3:40 (2:30-6:12)	0:05-85:10
Total fasting time (n= 283)	1,200 (1,010-1,500)	540-6,300	20:00 (16:50-25:00)	9:00-105:00
Fasting time - elective surgeries				
Preoperative (n=194)	688 (580-850)*	285-1600	11:28 (9:40-14:10)*	4:45-26:40
Postoperative (n=195)	205 (145-310)	5-5110	3:25 (2:25-5:10)	0:05-85:10
Total fasting time (n= 194)	1,220 (1,010-1,440)	540-6,210	20:20 (16:50-24:00)	9:00-103:30
Fasting time - urgent surgeries				
Preoperative (n=89)	760 (630-975)*	200-2,905	12:40 (10:30-16:15)*	3:20-48:25
Postoperative (n=89)	225 (160-760)	50-5,070	3:45 (2:40-12:40)	0:50-84:30
Total fasting time (n=89)	1,180 (1010-1950)	680-6,300	19:40 (16:50-32:30)	11:20-105:00

Comparison of fasting times between types of surgery (elective vs. urgency) - Wilcoxon rank-sum (Mann-Whitney) test: Total fasting p=0.649; Preoperative fasting p=0.025*; Postoperative fasting p=0.06. IQR: Interquartile Range.

Comparison of fasting times between types of surgery (elective vs. urgency) - Wilcoxon rank-sum (Mann-Whitney) test: Total fasting p=0.649; Preoperative fasting p=0.025*; Postoperative fasting p=0.06. IQR: Interquartile Range.

For all patients, the median fasting time was 11 hours and 40 minutes for the preoperative period, 3 hours and 40 minutes postoperatively, and 20 hours for total fasting (Table 3). Excessive preoperative fasting time also occurred when patients were stratified by type of food or liquid ingested (Table 4).

**Table 4:** Preoperative fasting time of patients admitted to the pediatric surgery ward of Hospital São Paulo (August/2020 to October/2021), according to the type of food.

	Median (IQR) (minutes)	Range (minutes)	Median (IQR) (hours:minutes)	Range (hours:minutes)
Type of food - all patients				
Full meal (n=250)	832 (680-1035)	420 - 6540	13:52 (11:20-17:15)	7:00 - 109:00
Clear liquids (n=247)	730 (630-940)	200 - 2905	12:10 (10:30-15:40)	3:20 - 48:25
Dairy (breast milk n=26)	445 (375-550)	285 - 840	7:25 (6:15-9:10)	4:45 - 14:00
Dairy (other milk n=71)	660 (565-780)	330 - 1630	11:00 (9:25-13:00)	5:30 - 27:10
Type of food - elective surgeries				
Full meal (n=179)	800 (670-1005)*	502 - 6540	13:20 (11:10-16:45)*	8:22 - 109:00
Clear liquids (n=176)	720 (630-908)*	360 - 1600	12:00 (10:30-15:08)*	6:00 - 26:40
Dairy (breast milk n=19)	395 (360-550)	285 - 840	6:35 (6:00-9:10)	4:45 - 14:00
Dairy (other milk n=45)	650 (555-750)	440 - 1630	10:50 (9:15-12:30)	7:20 - 27:10
Type of food - urgent surgeries				
Full meal (n=71)	900 (720-1440)*	420 - 2970	15:00 (12:00-24:00)*	7:00 - 49:30
Clear liquids (n=71)	800 (670-1000)*	200 - 2905	13:20 (11:10-16:40)*	3:40 - 48:25
Dairy (breast milk n=7)	480 (430-560)	380 - 760	8:00 (7:10-9:20)	6:20 - 12:40
Dairy (other milk n=26)	732 (600-885)	330 - 1310	12:12 (10:00-14:45)	5:30 - 21:50

Comparison of preoperative fasting times, divided by type of food, between the types of surgery (elective x urgent) - Wilcoxon rank-sum (Mann-Whitney) test: Preoperative fasting complete meal p=0.001*; Preoperative clear liquids fasting p=0.008*; Preoperative fasting: breast milk, p=0.183; Preoperative fasting: other milks, p=0.142. IQR: Interquartile Range.

The preoperative fasting time for elective surgeries was shorter than for urgent ones (Table 3). This result also appears in the analysis of clear liquids or full meals before surgery (Table 4).

The factors associated with preoperative fasting time that remained in the final linear model were age (p<0.001) and the occurrence of previous surgery (p=0.002), both adjusted for sex and type of surgery (elective or urgent). For each additional year of life, there was a 10-minute increase in fasting time (β=10, 95%CI 5.2-14.8). Previous surgery increased fasting time by 76.6 minutes (β=76.6, 95%CI 28.0-125.1) (Table 5).

**Table 5:** Simple and multiple linear regression of factors associated with preoperative fasting time, in minutes, of pediatric patients hospitalized and undergoing surgical procedure at Hospital São Paulo (August/2020 to October/2021).

Variables		Univariate Analysis			Univariate Analysis		
	N	β	95% CI	p-value	β	95% CI	p-value
Male	273	-44.8	-95.5 - 6.0	0.083	-34.3	-83.4 - 14.8	0.170
Age in years	273	11.8	7 - 16.5	<0.001	10	5.2 - 14.8	<0.001
ABEP score	271	-2.7	-6.3 - 0.8	0.134			
Maternal age in years	268	0.14	-3 - 3.3	0.931			
Maternal schooling in years of study (n=264)	264	-5.8	-15.1 - 3.5	0.218			
Paternal age in years (n=256)	256	0.78	-2 - 3.6	0.586			
Paternal schooling in years of study (n=228)	228	-6.8	-16.6 - 2.9	0.168			
Congenital pathological history	273	-1.4	-52.2 - 49.5	0.958			
Acquired pathological history	273	39.7	-10.2 - 89.6	0.118			
Prematurity	266	41.1	-20.2 - 102.5	0.188			
Birth weight in kilograms	240	-30.5	-65.5 - 4.2	0.085			
Previous surgery	273	93.9	45 - 142.8	<0.001	76.6	28.0 - 125.1	0.002
Elective surgery	273	-22.8	-77.7 - 32.2	0.416	-26.3	-78.6 - 25.9	0.322
ASA Rating	237	22.8	-18 - 63.5	0.273			
Preoperative site not the surgical ward	273	-21.5	-86.7 - 43.8	0.518			
Fasting guidelines conveyed the day before surgery	272	95.8	-62.6 - 254.2	0.235			
Fasting Guidelines conveyed in person	272	-68	-151.7 - 15.7	0.111			
Fasting guidelines conveyed by the surgeon in charge	272	-14	-64.3 - 36.4	0.586			
Anesthetic size	273	0.53	-17 - 18.1	0.952			
Abdominal surgery	273	68	-5.6 - 141.6	0.07			

β: Regression coefficient; 95% CI: 95% Confidence Interval.

The incidence of postoperative fasting time greater than six hours was 26.1% (95%CI=21.3-31.5). The factors that remained in the final logistic model associated with a higher risk of postoperative fasting of more than six hours were postoperative hospitalization not in the surgical ward (p<0.001), presence of intraoperative complications (p=0.023), surgical size III or IV (p=0.005), abdominal surgery (p<0.001), and presence of vomiting in the first 24 hours postoperatively (p=0.003), all adjusted for sex, age, and type of surgery (Table 6). Among these factors, abdominal surgery is particularly notable, presenting a risk that is more than 36 times higher. Additionally, the immediate postoperative period outside the surgical ward carries a risk that exceeds six times the baseline.

**Table 6:** Simple and multiple logistic regression of factors associated with prolonged postoperative fasting time (> 6 hours)* of pediatric patients hospitalized and undergoing surgical procedures at Hospital São Paulo (August/2020 to October/2021).

Variables		Univariate Analysis			Multivariate Analysis		
	N	OR	95%CI	P-value	OR	95%CI	P-value
Male	284	0.96	0.56 - 1.65	0.878	0.96	0.45 - 2.05	0.913
Age 5 years or younger	284	0.71	0.41 - 1.21	0.205	1.00	0.99 - 1.01	0.534
ABEP class C1, C2 or D-E	282	1.87	0.99 - 3.53	0.054			
Mother under the age of 20	279	1.13	0.21 - 5.97	0.883			
Maternal schooling 10 years or less (years of study)	275	1.80	1.04 - 3.13	0.036			
Father under the age of 20	267	2.75	0.38 - 19.9	0.315			
Paternal schooling 10 years or less (years of study)	238	1.37	0.77 - 2.42	0.279			
Congenital pathological history	284	0.75	0.44 - 1.27	0.282			
Acquired pathological history	284	1.04	0.61 - 1.76	0.897			
Prematurity	276	1.03	0.53 - 2.01	0.922			
Previous surgery	284	0.71	0.41 - 1.21	0.208			
Elective surgery	284	0.53	0.30 - 0.92	0.024	0.48	0.21 - 1.13	0.094
Variables		Univariate Analysis			Multivariate Analysis		
	N	OR	95%CI	P-value	OR	95%CI	P-value
Classification of ASA III, IV, V, or VI	247	1.79	0.74 - 4.31	0.195			
Postoperative site not the surgical ward	284	8.68	3.99 - 18.98	<0.001	6.05	2.25 - 16.22	<0.001
Intraoperative complications	284	3.86	1.78 - 8.38	0.001	3.53	1.19 - 10.47	0.023
Surgical size III or IV	283	3.93	2.07 - 7.48	<0.001	3.85	1.49 - 9.93	0.005
Anesthetic size greater than or equal to 5	284	1.60	0.94 - 2.73	0.084			
Abdominal surgery	284	27.47	11.39 - 66.28	<0.001	36.52	13.48 - 98.91	<0.001
Vomiting in the first 24 hours postoperatively		3.50	1.98 - 6.20	<0.001	3.44	1.54 - 7.69	0.003

*Incidence: 26.1% (95% CI: 21.3 -31.5); OR: Odds Ratio; 95%CI: 95% Confidence Interval.

## DISCUSSION

Prolonged fasting occurred both pre- and postoperatively, and for the preoperative period, all patients fasted longer than current recommendations^23^ ^-^ ^25^. 

The preoperative fasting time for elective surgeries was shorter than for emergency ones, especially when clear liquids and full meals were evaluated. This difference did not occur in postoperative fasting.

The factors associated with longer preoperative fasting time were older age in years and the occurrence of previous surgery. On the other hand, the factors associated with postoperative fasting time greater than six hours were the immediate postoperative period outside the surgical ward, the presence of intraoperative complications, larger surgical size, abdominal surgery, and the presence of vomiting in the first 24 hours postoperatively.

Although several scientific publications in recent years have reinforced the safety and benefits of adopting a preoperative fasting regimen with a shorter time, there are still difficulties in applying this approach in clinical practice, as demonstrated in our study^4^ ^-^ ^6^ ^,^ ^13^ ^,^ ^14^ ^,^ ^26^ ^,^ ^27^. This same finding was corroborated by other authors from different countries. Table 7 shows a trend towards shorter time in more recent studies, except in those carried out in non-developed countries.

**Table 7:** Preoperative fasting time according to the type of food and age group.

Author (Year)	Population - local	FT Residue-free liquids	FT Solid	FT breast milk
Pearse e Rajakulendran (1999)^28^	Adults (n=153) - England	12:30#	15:24#	-
Cestonaro et al. (2014)^29^	Adults (n=135) - Brazil	15:45*	16:30*	-
Gebremedhn e Nagaratnam (2014)^30^	Adults and children (n=43) - Ethiopia	12:43#	19:36#	-
Francisco et al. (2015)^27^	Adults (n=59) - Brazil	15:50*	16:00*	-
Williams et al. (2014)^25^	Children (n=219) - USA	10:26#	10:37#	08:18#
Dolgun et al. (2017)^31^	Children (n=332) - Turkey	10:31#	11:13#	06:16#
Al-Robeye et al. (2020)^32^	Children (n=71) - England	06:54#	11:42#	-
Kouvarellis et al. (2020)^33^	Children (n=585) -South Africa	08:00#	13:54#	07:06#
El-Sharkawy et al. (2021)^23^	Adults (n=343) - England	5:48*	16:06*	-
Assen et al. (2021)^34^	Children (n=258) - Ethiopia	12:19#	13:15#	07:45#
Yimer et al. (2022)^35^	Children (n=279) - Ethiopia	10:00#	13:30#	07:12#

*Median; #Average. Fasting time (FT) in the format hours:minutes (hh:mm).

The main hypothesis is that the excessively long time occurs due to the lack of an institutional protocol, the team’s concern about pulmonary aspiration during anesthesia, the care team with outdated beliefs, and the recurrent medical prescription of overnight fasting^27^ ^,^ ^29^ ^,^ ^33^ ^,^ ^36^ ^,^ ^37^. There is also a mistaken consensus that uniform prescription is more comprehensible, less likely to make mistakes, and facilitates possible changes in the surgical map, increasing the convenience of the entire team, which has not been proven in the literature^38^. 

The night fasting prescription stands out, being widespread and recurrent in most hospital services. In the present study, almost 80% of patients received overnight fasting guidance. Other studies have also found difficulty in breaking the paradigm of night fasting, evidencing frequent forms of prescription such as “fasting from midnight” or “fasting after 10 pm”^27^ ^,^ ^29^ ^,^ ^39^.

The excessively long time identified here can be explained by the routine of the São Paulo hospital, in which dinner is served at 6 pm and supper at 8:30 pm. After that time, only water or milk is available to patients. Despite this, the minority (4.1%) of elective surgeries had some specific recommendations for the consumption of clear liquid, milk formula, or breast milk after supper. Even for surgeries scheduled in the afternoon, approximately nine out of ten patients had overnight fasting in their prescription and remained fasting until surgery. Other study^29^ also reported the absence of guidance for the consumption of such foods. 

In services with a defined protocol, there are also difficulties in shortening fasting time, which can reach 19 hours for solids and eight hours for liquids^33^ ^,^ ^40^. Specifically, van Noort et al. (2021) demonstrated that approximately half of patients evaluated were instructed to “fast from midnight”, even though there was a protocol to be followed^36^.

Some hypotheses of non-adherence to the protocols include the way information about fasting is transmitted, the level of understanding on the part of the care team and patients, the patient being asleep early in the morning before being taken to the operating room, and delays regarding readiness of the surgical material and authorizations to perform the procedures. However, the main reason for these findings would be alterations in surgical map planning (delays and relocations), which happen frequently, even on ordinary days^6^ ^,^ ^24^ ^,^ ^35^. Approximately two-thirds of the elective surgeries we studied had a scheduled time on the surgical map. Of these, there was a delay in the onset of anesthesia of 60 minutes or more in most cases. Regardless of the surgical map, more than half of medical prescriptions for preoperative fasting did not follow the recommendation of the pre-anesthetic evaluation form. In this line, effective communication between medical specialties and delays and changes in schedules between teams of the operating room and wards would enable a reduction in fasting time^5^ ^,^ ^33^. 

Despite these difficulties, some authors have been able to demonstrate that when institutional protocols are adhered to, the preoperative fasting time of adult and pediatric patients is reduced, without necessarily increasing anesthetic complications and surgical cancellations, as desirable^39^ ^,^ ^41^ ^-^ ^44^. 

The estimated postoperative fasting time in our study was shorter than that found in most studies in the literature^27^ ^,^ ^29^ ^,^ ^45^. Early postoperative oral feeding can be challenging due to logistical issues within the nutrition service, including mismatched timing between medical and nutrition visits and limited flexibility in diet delivery schedules^29^. In addition, there has also been a fear of increased nausea and vomiting or worsening of ileus duration^46^.

In this sense, Schenk et al. (2022) managed to reduce the average time to resume postoperative diet by six hours with the implementation of a nutrition delivery service in the post-anesthetic care unit. In addition to greater patient satisfaction, earlier elimination of flatus was also evidenced and there was no higher incidence of nausea or vomiting^46^.

The shorter postoperative fasting time evidenced in the present study can be explained by the diversity of surgeries, which included low-complexity procedures (surgical size I and II) in most cases and, consequently, facilitated diet reintroduction. The presence of a specific hospitalization unit for the care of surgical cases in the pediatric age group, with a full-time medical team, may have contributed to better postoperative results.

Sun et al. (2022) found a lower postoperative diet reintroduction time than ours, probably due to an institutional early refeeding protocol in the service studied. Thus, the presence of updated protocols is also potentially capable of reducing postoperative fasting time for both low-grade and high-complexity surgeries^40^ ^,^ ^42^. 

Regarding the factors associated with prolonged preoperative fasting, we demonstrated that fasting time increases with age. This same finding was reported by Kouvarellis et al. (2020)^33^. The fasting time for breast milk is generally shorter than for other foods, as evidenced in our and other studies (Table 7). Younger children have breastfeeding readily available in addition to the habit of breastfeeding after midnight, which would potentially explain this association.

The occurrence of previous surgery was also a factor associated with longer preoperative fasting time in our study. This may be related to patients’ greater clinical complexity, which potentially generates greater concerns among the medical team with the shortening of fasting. Despite the plausibility of this association, we did not find studies in the literature that tested this hypothesis.

Patients admitted to the emergency room may spend long hours waiting for a vacancy in the operating room and therefore have a higher risk of long preoperative fasting. This association was evidenced in the literature^24^ ^,^ ^25^ and in the initial comparison made here. However, after the multiple linear regression model, this association did not remain statistically significant, as demonstrated in another study^23^.

We should note that information on the characteristics of variable distribution, sample normalization, and adjustment of multiple models has not been frequently presented by other authors who study preoperative fasting, and only tests to compare means and medians between groups are used, which may hinder the adequate interpretation of the effect of the type of surgery on fasting time^24^ ^,^ ^25^. Thus, further studies that consider multifactorial characteristics and control for confounding are needed to confirm or refute this finding. 

Among the factors associated with postoperative fasting time of more than six hours, the main one was abdominal surgery, as demonstrated in other studies^29^ ^,^ ^45^. Supposedly, in abdominal surgeries, the belief that the paralytic ileus time would be shorter when waiting for the gastrointestinal tract to function before resuming diet is even more relevant^27^.

The other factors associated with longer postoperative fasting (failure to perform the immediate postoperative period in the surgical ward, presence of intraoperative complications, and larger surgical size) are probably related to surgery complexity and patient severity, which slow diet reintroduction. Potentially, patients referred to the ICU, submitted to very long procedures or with some complication, have a longer postoperative fasting period, as they lack the clinical conditions to immediate diet restart. In addition, these patients are assisted by a medical team other than that of the specialized ward. Although these findings are clinically acceptable, these associations have not been investigated by other authors.

The most common complication in the first 24 hours postoperatively was vomiting, affecting approximately one in four patients. This clinical complication was also associated with longer postoperative fasting time. It was not possible to identify the temporal sequence of these two phenomena. Vomiting can delay the start of the diet, and the longer fasting time can lead to a higher incidence of postoperative vomiting, as evidenced in other studies41,45. These findings suggest that preventing vomiting reduces postoperative fasting time and that waiting longer for diet start does not reduce its occurrence as previously believed, as already demonstrated in the literature^16^ ^,^ ^47^ ^,^ ^48^. 

In our study, only four patients received written information about preoperative fasting, which made it difficult to make statistical comparisons between groups. However, there are reports that written instructions given to patients reduce preoperative fasting time^24^ ^,^ ^49^ ^,^ ^50^. 

We realize the importance of having institutional protocols for shortening fasting, which results in better postoperative recovery and greater comfort for pediatric patients. For their implementation, there is a need for instruction and training of the entire multidisciplinary team, homogeneity of prescriptions, and patient guidance. To this end, it seems important to create constant and effective communication measures among care teams, which may include the use of various tools, such as emails, classes, folders, meetings, etc. There is also need for collaboration from the hospital nutrition service to provide diet in the pre- and postoperative periods, when requested by the medical team. 

The present prospective cohort included enough pediatric patients hospitalized for surgical procedures of various complexities to find differences in the outcomes studied between groups. Regarding fasting times, the results found are consistent with the current world literature, which suggests that the same inadequacies found may occur in other scenarios. Interviews with caregivers were conducted to reduce the risk of recall bias, and patients and their families were not informed about the main objectives of this study, which reduced the risk of biases due to response induction. In addition, the option for multiple analysis to study associations made it possible to control confounding factors and identify independent effects.

On the other hand, some characteristics potentially associated with outcomes have not been studied, such as preoperative nutritional status, which may contribute to greater surgical complications, and intraoperative opioid use, which may contribute to longer ileus time. Also, the type of food ingested in the postoperative period was not evaluated separately, which made it difficult to estimate the caloric and protein intake ingested after surgery. Another point to highlight is that elective and urgent surgeries were studied during the COVID-19 pandemic, when we remained several weeks without elective hospitalizations, which may have influenced the proportion of the type of surgery and the care dynamics.

## CONCLUSION

In our study, all patients paused their diet in the preoperative period much earlier than the current recommendations, regardless of the type of diet. As for the recommendation to restart the diet on the first postoperative day, this goal, in most cases, was achieved.

Reduction of preoperative fasting time can be achieved when there is a multiprofessional institutional protocol, with training and organization of the team from different sectors, with special attention to older patients and those with previous surgery.

The creation of a protocol aimed at the postoperative period can facilitate early identification of patients at risk, such as those undergoing abdominal surgery or surgeries of larger size and the presence of intraoperative complications and, in this way, conduct care focused on optimizing diet reintroduction. Furthermore, for more expressive results to be achieved, this protocol could include early treatment of vomiting in the first 24 hours and training of the medical team and organization of ICUs.

Our results reinforce that, despite the growth of scientific knowledge in the area, there is still a considerable distance between recommendations about the abbreviation of fasting and clinical practice. Possible causes of this are multifactorial and dependent on the specific characteristics of the structure and dynamics of hospital care. Our findings may contribute to supporting institutional protocols that increase adherence to the recommendations, since it is already known that shortening surgical fasting is beneficial, safe, and plausible. 
